# Fruits and Seeds as Indicators of the Genetic Diversity of *Hymenaea martiana* (Fabaceae) in Northeast Brazil

**DOI:** 10.3390/biology14101418

**Published:** 2025-10-15

**Authors:** Joyce Naiara da Silva, Guilherme Vinícius Gonçalves de Pádua, Caroline Marques Rodrigues, João Henrique Constantino Sales Silva, Cosma Layssa Santos Gomes, Marília Hortência Batista Silva Rodrigues, Maria Karoline Ferreira Bernardo, Eduardo Luã Fernandes da Silva, Luís Gustavo Alves de Almeida, Lenyneves Duarte Alvino de Araújo, Aline das Graças Souza, Naysa Flávia Ferreira do Nascimento, Edna Ursulino Alves

**Affiliations:** 1 Department of Plant Science and Environmental Sciences, Center for Agrarian Sciences, Federal University of Paraíba, Campus II, Areia 58397-000, PB, Brazil; marxcarol48@hotmail.com (C.M.R.); layssasnts@gmail.com (C.L.S.G.); karolinebernardo249@gmail.com (M.K.F.B.); eduardo.eng.fernandes@gmail.com (E.L.F.d.S.); luis.alves2@academico.ufpb.br (L.G.A.d.A.); alinedasgracas@yahoo.com.br (A.d.G.S.); naysa.flavia@academico.ufpb.br (N.F.F.d.N.); ursulinoalves@hotmail.com (E.U.A.); 2 Federal Institute of Education, Science and Technology of Amazonas, Coari Campus, Coari 69460-000, AM, Brazil; guilhermegpadua@yahoo.com.br; 3 Center for Agrofood Science and Technology, Federal University of Campina Grande, Pombal 58840-000, PB, Brazil; mariliahortencia8@gmail.com; 4 Department of Biosciences, Center for Agrarian Sciences, Federal University of Paraíba, Campus II, Areia 58397-000, PB, Brazil; lenyneves@academico.ufpb.br

**Keywords:** conservation, jatobá, phenotypic markers, seed analysis, variability

## Abstract

**Simple Summary:**

Genetic diversity is crucial for species’ resilience to climate change and for conservation. *Hymenaea martiana*, a native species with significant ecological and socioeconomic value, was evaluated for fruit and seed variability and seed physiological quality. We analyzed 160 mother plants and, through several comparisons, identified eleven distinct groups, which demonstrated significant variability. Among the descriptors studied, seed length and weight, the emergence speed index, and seedling shoot dry mass were the most important contributors to individual differentiation. These results reinforce the importance of maintaining genetic diversity in *H. martiana*, especially in the context of climate change, ensuring evolutionary resilience and supporting future sustainable use programs. This study contributes to the genetic management of this species and the enrichment of germplasm banks, which are essential for biodiversity conservation and the valorization of genetic resources in tropical forests.

**Abstract:**

*Hymenaea martiana* is a species native to Brazil. It has ecological value, contributes to forest restoration, and is economically important because of the use of its wood and fruits. However, it is frequently exploited. Therefore, understanding genetic diversity becomes essential for guiding conservation strategies as well as ecological restoration actions in the face of climate change and anthropogenic pressures. Thus, this study aimed to evaluate the intraspecific diversity of 160 *H. martiana* mother plants on the basis of morphological descriptors of fruits and seeds and physiological indicators of seed quality, identifying the most discriminating characters. Eighteen traits were analyzed and subjected to analysis of variance and the Scott–Knott test (*p* < 0.05), with estimates of heritability and the ratio between genetic and environmental coefficients of variation. Phenotypic divergence was obtained via the Mahalanobis distance (D^2^) and grouped via UPGMA, whereas the relative contribution of the traits was estimated via the Singh method. The results revealed that seed length and weight, emergence speed index, and shoot dry mass were the most effective descriptors for discriminating parent plants. Multivariate analysis revealed the formation of eleven phenotypically distinct groups, demonstrating high variability. These findings support the selection of superior genotypes and representative seed collection, as well as practical initiatives such as the formation of germplasm banks, the selection of breeding stock for forest nurseries, and reintroduction programs. Thus, the data obtained offer technical and scientific support for biodiversity conservation and ecosystem recovery in the semiarid region of Brazil.

## 1. Introduction

The genus *Hymenaea* L. (Fabaceae) comprises 18 species, of which 12 are endemic to Brazil [1]. In Brazil, especially in the North, Northeast, Central–West, and Southeast Regions, the species *Hymenaea courbaril* L., *Hymenaea stigonocarpa* Mart., and *Hymenaea martiana* Hayne stand out economically [2,3]. The *H. martiana* species has a geographic distribution restricted to Brazil, Argentina, Bolivia, Colombia, and Paraguay, occurring in ecosystems such as the Caatinga, Cerrado, and Atlantic Forests. It is a tree that can reach 8 to 18 m in height, with dense wood used in construction [4,5,6].

In addition to its timber value, *H. martiana* has multiple uses, such as the bark and resin of the plant, which are used in folk medicine to treat illnesses such as bronchitis, anemia, gastritis, inflammation, rheumatism, injuries and gastric disorders [7,8]. The floury pulp of the fruit has nutritional potential and can replace wheat flour [9]. Recent studies also point to the photoprotective properties associated with flavonoids present in the stem bark [10]. Although the IUCN [5] classifies *H. martiana* as ‘Least Concern’, the disorderly exploitation of plant resources, especially in biomes such as the Caatinga, has generated growing concerns about the conservation of the species [11].

Genetic variability is essential for the adaptation of species to environmental pressures and climate change [12,13]. Its reduction compromises the resilience of populations and limits improvement programs [14]. Therefore, conservation strategies must be supported by robust information on available diversity, integrating in situ and ex situ actions [15,16].

In this sense, different approaches have been employed to characterize diversity, including molecular and biochemical techniques and the analysis of phenotypic or morphological characteristics [15,17]. Among these, the use of phenotypic attributes stands out because of their practicality, lower cost, and ease of measurement, and they do not require sophisticated equipment. In forest species such as *Quercus variabilis* Blume and *Leucaena leucocephala* (Lam.) de Wit, these descriptors have already been successfully used to identify intrapopulation variability and guide sustainable management strategies [18,19]. These examples demonstrate that morphological characterization is a widely applied tool in forest species, reinforcing its relevance for *H. martiana*.

In *H. martiana*, the morphological attributes of fruits and seeds, in addition to the vigor of seeds and seedlings, are important indicators for the selection of mother plants since propagation occurs mainly via seeds [20]. These largely hereditary characteristics reinforce the need to monitor individuals with superior potential to ensure their transmission to progeny. Selection should consider not only dendrometric and dendrological criteria but also attributes related to the physical and physiological quality of the seeds [21,22]. Genetic variability expressed by the morphometric characteristics of fruits and seeds is useful in studies of intrapopulation divergence, providing support for genetic improvement and for collection and conservation strategies [23].

Despite the socioeconomic importance of *H. martiana*, few studies have investigated its diversity on the basis of phenotypic attributes. This gap hinders the definition of effective conservation strategies, especially in regions such as Northeast Brazil, where intensifying droughts and inadequate land use exacerbate environmental pressures. Thus, although it does not utilize molecular markers, phenotypic characterization can reveal population structure and indicate useful traits for selection and conservation programs.

Therefore, this study evaluated the intraspecific genetic diversity in 160 *H. martiana* mother plants on the basis of the morphological characteristics of the fruits and seeds, in addition to the physiological quality of the seeds, identifying the characters with the greatest contribution to this divergence on the basis of univariate and multivariate descriptors.

## 2. Materials and Methods

### 2.1. Collection Location

*H. martiana* fruits were collected from 160 mother plants (Table 1). The mother plants were randomly selected because of their good phytosanitary status. Collections were made when the fruits were ripe, using the dark brown color of the fruits and their dehiscence from the trees as morphological markers. The fruits were collected from under the tree canopy after they had fallen.

After collection, the samples were subsequently sent to the Seed Analysis Laboratory of the Department of Plant Science and Environmental Sciences, Center of Agricultural Sciences of the Federal University of Paraíba (DFCA–CCA/UFPB) for physical and physiological analyses.

### 2.2. Physical Characterization of Fruits and Seeds

For the physical characterization of the fruits, 60 units per mother plant were used (four replicates with 15 fruits), and the length, width, and thickness were evaluated via a digital caliper (0.01 mm accuracy) (ZAAS Precision, Ultracorte Tools Trading LTDA, São Paulo, SP, Brazil). The weight was obtained via a semi-analytical balance with a precision of 0.001 g (Bioprecisa-JA3003N, Bioprecisa, Curitiba, PR, Brazil). To determine the number of seeds per fruit, the fruits were broken open with a wooden stick, and then the seeds were counted. The seeds were subsequently processed according to Silva’s methodology [29].

For the physical characterization of the seeds, 100 seeds were used per mother plant (four replicates of 25 seeds), and the length, width, and thickness were measured with a digital caliper (accuracy of 0.01 mm). A precision analytical balance (0.001 g) was used to obtain the unit weight of the seeds.

The moisture content of the seeds was determined via the oven method at 105 ± 3 °C for 24 h [30], with four replicates of 10 ± 0.5 g of seeds per mother plant. The results are expressed as percentages.

### 2.3. Seedling Emergence Test

Prior to test installation, the seeds were scarified due to tegumentary dormancy. The procedure was performed on the side opposite the hilum via iron sandpaper No. 120, according to Silva [29]. Then, superficial disinfection of the seeds was carried out in a 1% sodium hypochlorite solution for 3 min [31].

The plants were seeded in plastic pots measuring 30 cm in diameter × 22 cm in height. These samples were filled with medium-grain sand previously sterilized in an autoclave at 120 °C for 120 min [30]. A total of 5 cm was left free to the edge. Each pot received four replicates of 25 seeds per mother plant, with a sowing depth of 3.0 cm.

The experiment was conducted in a greenhouse. The temperature and humidity were monitored daily with a thermohygrometer, which recorded an average temperature of 32 °C and a relative humidity of 60%. Substrate moisture was maintained by manual watering, which was performed daily as needed.

Emergence was considered when the cotyledons completely broke through the substrate surface, with the results expressed as a percentage. The first emergence count (FEC) was performed 25 days after sowing.

The emergence speed index (ESI) was calculated according to the equation proposed by Maguire [32], taking into account daily counts up to 33 days after sowing. The mean emergence time (MET) was calculated via the formula of Edmond and Drapala [33], and the results are expressed in days.

### 2.4. Initial Growth and Seedling Biomass

After completion of the emergence test, the seedlings were removed from the substrate to determine their length and dry mass. The shoot length (SL) was measured from the collar to the apical meristem, and the root system (RSL) length was measured from the collar to the tip of the main root using a ruler graduated in centimeters. The results are expressed in cm.

After the measurements, the shoot and root systems of each replicate were separated with scissors and placed in prelabeled Kraft paper bags. The samples were placed in a forced-air circulation oven at 65 °C for 72 h [29] to obtain dry mass. After drying, the dry masses of the aerial part (DMAP) and the root (DMR) were weighed on an analytical balance with an accuracy of 0.001 g, and the results are expressed in g seedling^−1^.

### 2.5. Experimental Design and Statistical Analysis

#### 2.5.1. Univariate Analysis

The experimental design was completely randomized (CRD), with four replicates. The data were subjected to Shapiro-Wilk normality tests, and the Bartlett test was used for homoscedasticity. Because the data were normally distributed, analysis of variance (ANOVA) was performed. When significant, the means were grouped via the Scott–Knott test at 5% probability. This test was chosen because it groups the means into homogeneous classes, facilitating the interpretation of results in experiments with many treatments. Statistical analyses were performed via R software version 4.2.1 [34] via the ScottKnott package [35].

#### 2.5.2. Genetic Parameters

Genetic parameters and their estimators were analyzed for each trait via the following mathematical expressions [36]:

(a) Phenotypic variance: ${\hat{\sigma }}_{f}^{2}=\frac{{QM}_{g}}{k}$
(b) Environmental variance: ${\hat{\sigma }}_{e}^{2}=\frac{{QM}_{r}}{k}$
(c) Genetic variance: ${\hat{\sigma }}_{g}^{2}=\frac{{QM}_{g}-{QM}_{r}}{k}$
(d) Heritability in the broad sense: $h^{2}=\frac{{\hat{\sigma }}_{g}^{2}}{{\hat{\sigma }}_{f}^{2}}$
(e) Coefficient of genetic variation: ${CV}_{g}=\frac{\sqrt{\sigma_{g}}}{m}\times 100$
(f) Coefficient of environmental variation: ${CV}_{e}=\frac{\sqrt{\sigma_{r}}}{m}\times 100$
(g) Ratio $\frac{{CV}_{g}}{{CV}_{e}}$

These analyses were performed via Genes software (Version 1990.2023.15) [37].

#### 2.5.3. Genetic Diversity

The genetic diversity analysis used a measure of dissimilarity on the basis of the generalized Mahalanobis distance (D^2^) [38]. On the basis of this distance, the criterion proposed by Singh [39] was used to quantify the relative contribution of each trait to the genetic variability between the parent plants.

Cluster analysis was performed to describe the genetic divergence between the parent plants and aid in the identification of superior genotypes. The unweighted pair group method with arithmetic mean (UPGMA) method is a hierarchical method that calculates the distance between groups by the arithmetic mean of the distances between all pairs, graphically representing the similarity relationship between individuals [40]. This method is suitable for experiments with multiple genotypes, as it forms clear and easy-to-interpret hierarchical dendrograms. The determination of the cutoff point of the generated dendrogram, as well as the definition of the number of groups, were estimated on the basis of the Mojena [41] method, which considers the relative size of the fusion levels (distances) observed in the dendrogram.

Statistical analyses were performed via Genes software (Version 1990.2023.15) [37] and R version 4.2.1 [34] via the candisc [42], biotools [43] and factoextra [44] packages.

## 3. Results

### 3.1. ANOVA and Genetic Parameters

There was a significant effect at 1% probability for the 18 traits related to the physical characterization of fruits, seeds and their physiological quality, indicating variability in these traits among *H. martiana* mother plants. This heterogeneity reinforces the potential of the species for selection and conservation programs on the basis of morphological and physiological traits (Table 2).

Heritability values were above 85% for all the traits analyzed, except for seed thickness (71.66%), suggesting strong genetic control of the evaluated traits. The ratio between the genetic and environmental coefficients of variation (CVg/CVe) was greater than 1.0 for most variables, indicating a predominance of genetic variation over environmental variation under the study conditions. Seed length and weight stood out, with CVg/CVe ratios of 4.01 and 3.80, respectively. Seed thickness (0.79) and the emergence speed index (0.97) were lower, which may reflect a greater influence of environmental factors or a lower phenotypic range for these traits. (Table 1). Taken together, the results indicate that the evaluated traits are useful for identifying variability among *H. martiana* individuals.

### 3.2. Fruit Characterization

Marked differences were observed between the mother plants in terms of fruit size and weight, as well as the number of seeds per fruit (Table 3). The fruit length ranged from 59.42 mm to 153.27 mm. The width ranged from 26.90 mm to 62.50 mm, and the thickness ranged from 14.1 to 39.8 mm.

Weight, a variable highly associated with pulp yield, ranged from 17.94 g to 140.92 g. Mother plants such as 23, 68, and 153 produced fruits with greater mass, demonstrating their potential for commercial purposes. The average number of seeds per fruit ranged from two to nine among the mother plants studied, with a highlight on mother plant 23 (Table 3).

Among the individuals evaluated, mother plants 23, 68, and 74 stood out, having the highest average values across multiple traits. These mother plants represent genotypes with superior potential for commercial use and conservation, especially in programs targeting larger, heavier fruits with a greater number of seeds.

### 3.3. Physical Characterization of Seeds

The *H. martiana* seeds widely varied in size, weight, and water content among the 160 mother plants evaluated (Table 4). Length ranged from 18.9 to 29.7 mm, width from 15.8 to 25.8 mm and thickness from 10.1 to 22.4 mm, although the latter showed less differentiation between groups.

Seed weight (Table 4) varied widely, ranging from 2.62 g to 6.84 g. This diversity suggests the existence of genotypes with greater potential for producing robust seeds, possibly associated with reserve accumulation. The water content varied between 5.73% and 16.96%. Despite the influence of environmental factors, this trait also showed significant interindividual variation.

Overall, the results revealed significant phenotypic diversity among individuals, especially in terms of seed length, weight, and width. These variables can be used as auxiliary criteria in the selection of superior matrices, with emphasis on the seeds of matrices 5, 21, and 35, which presented the largest sizes.

### 3.4. Physiological Quality of Seeds and Seedling Performance

*H. martiana* seeds presented wide variability in terms of seedling vigor and performance (Table 5). The first emergence count varied from 0 to 94%, demonstrating high heterogeneity among the seeds from the mother plants. The final emergence percentage ranged from 8% to 96%, with 67% of the seeds from the mother plants evaluated achieving an emergence rate greater than 73%, indicating good viability for most individuals.

The emergence speed index ranged from 0.09 to 1.24, while the average emergence time ranged from 16 to 30 days (Table 5). These variations suggest the existence of more vigorous and efficient genotypes in mobilizing reserves, which may reflect an adaptive advantage.

The initial growth of the seedlings varied substantially (Table 5). The length of the aerial parts varied from 4.72 cm to 27.42 cm, whereas the length of the root system varied between 8.00 cm and 24.72 cm. The dry mass also reflected this diversity, with shoot dry mass ranging from 0.23 to 16.12 g seedling^−1^, whereas the root dry mass ranged from 0.15 to 1.32 g seedling^−1^. These data indicate that some mother plants produce seeds with a greater capacity to form vigorous seedlings, which can be decisive in natural environments or in environmental restoration programs.

Mother plants 23, 34, 103, and 120 presented higher values for multiple variables, demonstrating greater vigor and initial development. These results indicate that this species possesses useful variability for selecting mother plants with potential for natural regeneration or ecological restoration programs.

### 3.5. Cluster Analysis and Contribution of Traits

**Figure 1:**
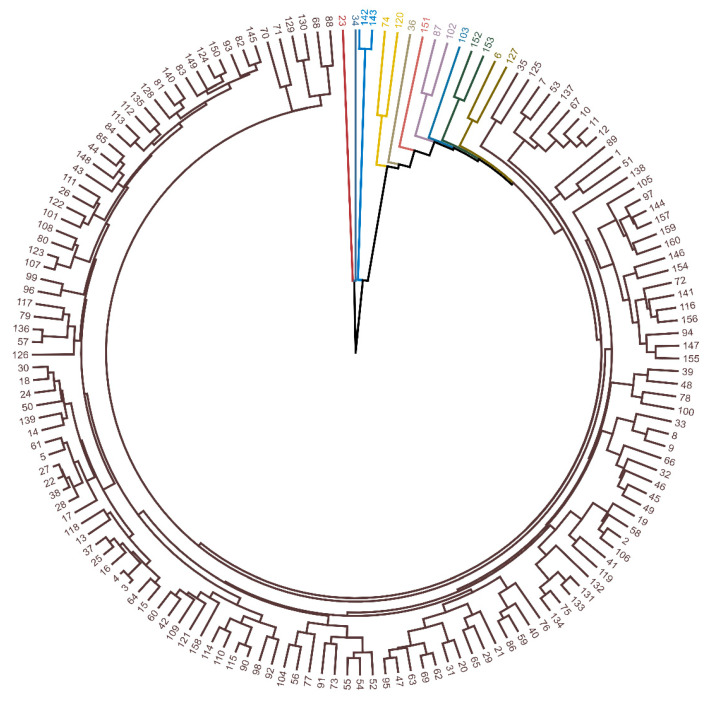
Dendrogram representing the pattern of dissimilarity among the 160 mother plants of *Hymenaea martiana* obtained via the UPGMA method from 18 variables related to the physical characterization of fruits and seeds and the physiological quality of the seeds. Different colors indicate distinct groupings.

UPGMA cluster analysis (Figure 1), which is based on the generalized Mahalanobis distance, revealed 11 distinct groups among the 160 *H. martiana* parent plants (Figure 1). The cophenetic correlation coefficient was 0.93, indicating high representativeness of the dendrogram in relation to the dissimilarity matrix. Using the Mojena [41] criterion with a cutoff of 2.1, a well-defined cluster structure was identified, with a predominance of group G9, which contained the majority of individuals (90.6%).

Five groups were formed by a single mother plant, which presented highly distinct phenotypic profiles: mother plant 23 (G10), with large, heavy fruits and vigorous seedlings; mother plant 34 (G11), with high shoot dry mass; mother plant 36 (G3), with heavier, thicker seeds; mother plant 103 (G6), with greater root development; and mother plant 151 (G4), with wider fruits. These extreme individuals represent genotypes with unique characteristics and strategic potential for selection.

Other smaller groups (G1, G2, G5, G7, and G8) brought together pairs of individuals with relevant phenotypic similarities. Notably, pairs 74 and 120 (G2) presented shorter average emergence times and greater initial seedling vigor, respectively, than 152 and 153 (G7) did, whose fruit sizes were larger than the overall average.

**Figure 2:**
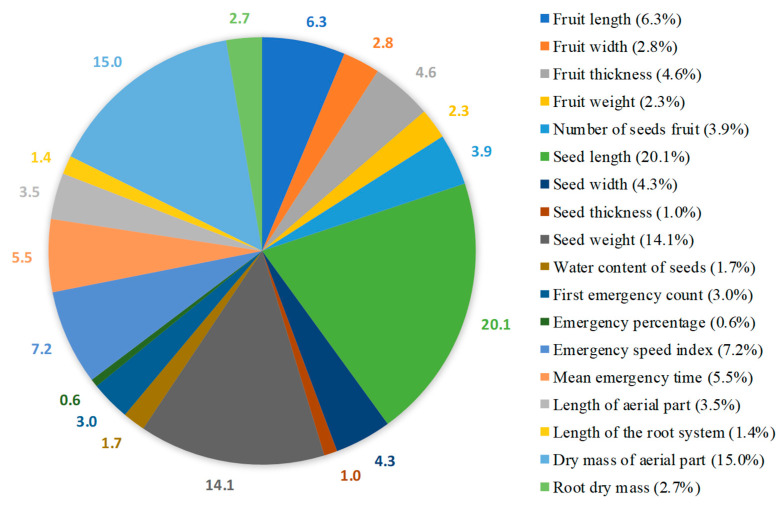
Estimates of the relative contribution of each variable to genetic divergence are based on the calculation of Mahalanobis distances according to Singh [39] criterion.

The analysis of the relative importance of the variables (Figure 2), according to the method of Singh (1981) [39], indicated that five characteristics explained 62.7% of the observed dissimilarity. Among the variables studied, seed length (20.1%) was the most discriminating variable, followed by shoot dry mass (15.0%), seed weight (14.1%), emergence speed index (7.2%) and fruit length (6.3%). In contrast, the variables that contributed least to the divergence were fruit thickness (1.0%) and seedling emergence percentage (0.6%).

These results reinforce the usefulness of multivariate approaches to identify individuals with superior phenotypic combinations, in addition to assisting in the selection of key variables for future studies and for the definition of management, conservation and improvement strategies.

## 4. Discussion

### 4.1. Analysis of Phenotypic Variability and Genetic Parameters

This study demonstrated high intraspecific genetic diversity in 160 *H. martiana* stock plants on the basis of 18 morphophysiological variables of fruits and seeds. The statistical significance observed for all variables reinforces the intraspecific heterogeneity of the species, indicating the potential for selection of superior stock plants and for the conservation of genetically diverse populations. This intraspecific heterogeneity is a valuable resource for conservation programs, as genetically more diverse populations tend to be more resilient to environmental stresses and climate change [17].

The high heritability estimates (>70%) and the ratio between the genetic and environmental coefficients of variation (CVg/CVe) above 1.0 for most traits point to the predominance of genetic variability under the study conditions, enabling genetic gains by selecting parent plants with superior phenotypic traits [45]. These results suggest that traits such as fruit weight, seed weight, and the emergence speed index may respond positively to selection, facilitating the identification and maintenance of superior genotypes [19,46]. These findings demonstrate that phenotypic descriptors represent viable tools for identifying genetic variability but do not completely replace molecular approaches.

### 4.2. Fruits and Seeds as Sources of Diversity and Added Value

The observed variation in fruit size and weight, as well as in the number of seeds per fruit, highlights the economic and ecological role of these attributes. Individuals with larger and more productive fruits can be strategic for extractive communities, reinforcing the added value of a species. When quantifying the phenotypic diversity of *Q. variabilis* fruits and their response to climatic factors, Gao et al. [18] reported a significant difference between and within 43 natural populations and reported that this diversity was closely related to local climatic factors such as temperature and precipitation.

The physical characteristics of the seeds also showed high heterogeneity. Previous studies, such as those by Menegatti et al. [47] on *Mimosa scabrella* Bentham, Bezerra et al. [48] on *Erythrina velutina*, and Willd and Wang et al. [49] on *Eucommia ulmoides*, also highlighted this divergence between the parent plants.

These results confirm that the biometric traits of fruits and seeds are practical indicators of genetic variability and contribute to understanding both dispersal dynamics and seedling establishment in native forests. According to Macedo et al. [50] and Gonçalves et al. [51], such descriptors allow not only the distinction between species of the same genus but also the identification of intrapopulation genetic variability, as well as the understanding of the influence of environmental factors on this variability.

### 4.3. Seed Vigor and Early Seedling Performance

One way to assess the physiological potential of seeds is to compare the vigor of seeds from mother plants of the same species because vigorous seeds produce individuals with a greater capacity to form a deep root system, which increases their adaptation to the environment [52,53]. Selecting more vigorous mother plants is therefore essential for producing resilient individuals.

Vigor analysis revealed that seeds from mother plants such as 74, 138, and 141 produced seedlings with faster emergence and greater shoot length. Vigorous seeds tend to germinate more quickly and efficiently, promoting greater mobilization of reserves to the embryonic axis, which favors the formation of a more efficient root system. This favors seedling survival in the early stages, especially in adverse environments [54].

Although the results reinforce the use of physiological parameters as phenotypic descriptors of genetic diversity, it is important to recognize that vigor can undergo seasonal and environmental variations, especially in semiarid regions. Thus, the data presented here strengthen, but do not exhaust, the use of these descriptors for inferring genetic variability.

### 4.4. Phenotypic Grouping

UPGMA cluster analysis has proven to be an effective tool for representing genetic divergence on the basis of phenotypic characteristics among *H. martiana* parent plants, enabling the formation of distinct groups on the basis of multiple morphophysiological traits. In practice, this approach allows for the selection of individuals with greater divergence, which is strategic for breeding programs, as it maximizes variability among crossed genotypes and, consequently, the potential for genetic gain [15]. Furthermore, the identification of parent plants that form isolated or highly differentiated groups indicates the presence of genotypes with rare or unique characteristics, which should be prioritized in in situ and ex situ conservation efforts.

The cophenetic correlation coefficient, in turn, revealed a strong correlation between the original distances and the generated dendrogram, validating the consistency of the cluster structure [55]. These results reinforce the use of UPGMA as a support tool in the selection of matrices representative of intraspecific variability.

Similar results were reported by Silva et al. [45], who, when evaluating 45 mother plants of *Parkia platycephala* Benth (Fabaceae), identified the formation of ten distinct groups via UPGMA and recommended the collection of seeds from plants belonging to different groups as a way to maximize genetic gains. Thus, the groupings obtained in both studies demonstrate the potential of the method as an auxiliary tool in the conservation of genetic variability and the composition of more representative germplasm banks.

Genetic diversity plays an important role in the stability and survival of populations, increasing the adaptability of genotypes to biotic and abiotic stresses. In the face of climate change, populations with broad genetic variability tend to have greater adaptive resilience, whereas those with reduced diversity are more vulnerable [17].

### 4.5. Characteristics Determining Genetic Divergence

The main objective of assessing the relative importance of traits in genetic divergence is to identify those with the least influence among the analyzed genotypes [40]. The variables fruit thickness and emergence percentage had the lowest contributions. However, although emergence percentage is a trait with a low contribution to this diversity, it is important for ecological or productive success, as it requires the use of seeds with a high germination/emergence percentage.

Similar results regarding the variability in the contributions of morphological traits have been reported in other species. In accordance with the methods of Singh [39], Correa et al. [56] reported that the traits with the greatest contribution to variability in Parkia pendula were fruit length, width, and thickness; seed width; and fruit mass. Similarly, when analyzing the genetic diversity of 22 quixabeiras (*Sideroxylon obtusifolium* [Humb. ex Roem. & Schult.] T.D. Penn.), Alves et al. [57] found that the seed length/diameter ratio, seed length, and fruit length were the most discriminative factors. These findings reinforce the usefulness of this approach in selecting more efficient descriptors to represent phenotypic variability among genotypes.

Identifying key traits strengthens the selection of more efficient descriptors and streamlines evaluation protocols in conservation and breeding programs. This approach allows the integration of phenotypic analysis into forest management and ecological restoration.

### 4.6. Implications for Conservation and Sustainable Use

The genetic diversity observed among *H. martiana* matrices reinforces the need to adopt conservation strategies that consider phenotypic performance. Plants with desirable traits and belonging to distinct groups should be prioritized for seed collection, which favors the formation of more heterogeneous and resilient seed lots [58].

According to Erickson & Halford [59], a seed lot is suitable for restoration purposes when its genetic diversity, which is representative of the source population, is preserved to the extent possible throughout the supply chain and is implanted at a restoration site with suitable ecological conditions. This diversity ensures the adaptive capacity of species in the face of climate change and different types of stress, both biologically and in an environmentally friendly manner [15]. In this context, the management of seed production units, the structuring of germplasm banks and the selection of matrices for restoration programs have become complementary and necessary strategies [60,61].

Identifying superior genotypes on the basis of accessible phenotypic attributes strengthens sustainable management practices and the ability to restore degraded ecosystems. Furthermore, it guides public policies and integrated local actions to increase ecological resilience in semiarid regions. This approach is aligned with the global goal of protecting biodiversity and promoting the sustainable use of natural resources. Despite methodological limitations, such as the absence of molecular markers and environmental influence, the results of this study indicate that phenotypic descriptors are effective tools for inferring genetic diversity in *H. martiana*.

In summary, three main messages emerge: (i) phenotypic descriptors reveal relevant genetic variation in *H. martiana*; (ii) the identified diversity has direct applications in conservation, breeding, and restoration; and (iii) these descriptors should be integrated into complementary approaches, recognizing methodological and environmental limitations.

## 5. Conclusions

The results suggest that morphophysiological descriptors of fruits and seeds, such as seed length and weight, the emergence speed index, and shoot dry mass, are effective indicators of intraspecific genetic diversity in *H. martiana*. This approach allows the identification of promising genotypes and the formation of phenotypically distinct groups, which is essential for the management and conservation of genetic resources.

The practical applicability lies in selecting matrices with superior performance in morphophysiological attributes and belonging to different groupings, ensuring greater genetic heterogeneity in nurseries and gene banks. Although the results support conservation and sustainable use strategies, in line with SDGs 13 and 15, it is essential to recognize limitations, such as the lack of molecular markers and the potential influence of environmental factors. Future studies should cover a larger number of populations and integrate genetic analyses to deepen the understanding of the diversity of this species.

## Figures and Tables

**Table 1:** Information on the collection areas for *Hymenaea martiana* fruits in the states of Paraíba and Rio Grande do Norte in Northeast Brazil.

Mother Plants	Collection Location	Coordinates	Region	Precipitation (mm year^−1^)	Average Temperature (°C)
1 to 136	Areia—PB	6°57′46″ S 35°41′31″ W	Brejo	>1400	21–25
137 to 140	Bananeiras—PB	6°45′0″ S 35°37′58″ W	Brejo	>1400	21–25
141	Cuité—PB	6°29′6″ S 36°9′25″ W	Western Curimataú	<400	>26
142 to 143	Marcação—PB	6°46′12″ S 35°0′54″ W	North coastline	until 1800	26
144 to 156	Nova Floresta—PB	6°27′18″ S 36°12′10″ W	Western Curimataú	<400	>26
157 to 160	Jaçanã—RN	6°25′33″ S 36°12′18″ W	Borborema Potiguar	500–800	25.6

Source: Adapted from Francisco & Santos [24], Arruda et al. [25], Nascimento et al. [26]; Beltrão et al. [27]; Nascimento et al. [28].

**Table 2:** Summary of the results of the analysis of variance of the physical characterization of fruits and seeds and the physiological quality of *Hymenaea martiana* seeds from 160 mother plants.

Sources of Variation	Mean Squares ^1^					
	FL (mm)	FWi (mm)	FT (mm)	FWe (g)	NSF	SL (mm)
Mother plants	837.33 **	184.07 **	99.76 **	1487.68 **	5.55 **	41.07 **
Residue	25.52	10.14	4.65	93.24	0.33	0.62
h^2^ (%)	96.95	94.49	95.34	93.73	94.06	98.47
CVg/CVe	2.82	2.07	2.26	1.93	1.99	4.01
CV (%)	5.54	7.29	7.53	15.61	17.08	3.23
**Sources of Variation**	**Mean Squares ^1^**					
	**SWi (mm)**	**ST (mm)**	**SWe (g)**	**WCS (%)**	**FEC (%)**	**EP (%)**
Mother plants	8.89 **	10.84 **	6.33 **	15.07 **	2579.24 **	1775.88 **
Residue	0.62	3.07	0.11	2.27	125.08	144.89
h^2^ (%)	92.95	71.66	98.29	84.91	95.15	91.84
CVg/CVe	1.81	0.79	3.80	1.19	2.21	1.67
CV (%)	4.15	12.51	6,57	13.49	26.61	20.48
**Sources of Variation**	**Mean Squares ^1^**					
	**ESI**	**MET (days)**	**LAP (cm)**	**LRS (cm)**	**DMAP** **(g seedling^−1^)**	**RDM** **(g seedling^−1^)**
Mother plants	0.29 **	32.55 **	61.09 **	39.28 **	7.84 **	0.08 **
Residue	0.02	1.00	3.81	5.82	0.05	0.009
h^2^ (%)	93.72	96.92	93.75	85.17	87.24	87.99
CVg/CVe	0.97	2.80	1.93	1.19	1.31	1.35
CV (%)	20.50	4.28	11.02	16.12	26.28	29.81

^1^ Significant effect at 1% (**) according to the F test. Legend: Heritability (h^2^); ratio between genetic and environmental coefficient of variation (CVg/CVe); coefficient of variation (CV); fruit length (FL); fruit width (FWi); fruit thickness (FT); fruit weight (FWe); number of seeds per fruit (NSF); seed length (SL); seed width (SWi); seed thickness (ST); seed weight (SWe); water content of seeds (WCS); first emergence count (FEC); emergence percentage (EP); emergence speed index (ESI); mean emergence time (MET); length of aerial part (LAP); length of the root system (LRS); dry mass of aerial part (DMAP); and root dry mass (RDM).

**Table 3:** Descriptive statistics of physical traits of *Hymenaea martiana* fruits from 160 mother plants.

Variable	Minimum	Maximum	Average ± SD	Scott–Knott Groups (5%)
Fruit length (mm)	59.4	153.3	91.12 ± 14.47	a–k
Fruit width (mm)	26.9	62.5	43.67 ± 6.78	a–h
Fruit thickness (mm)	14.1	39.8	28.61 ± 17.94	a–g
Fruit weight (g)	17.9	140.9	61.84 ± 19.28	a–h
Number of seeds per fruit	1.0	9.0	3.40 ± 1.14	a–f

SD: standard deviation. Note: Detailed values of the matrices by Scott–Knott group are available in the Appendix A.1 (Table A1).

**Table 4:** Descriptive statistics of physical traits of *Hymenaea martiana* seeds from 160 mother plants.

Variable	Minimum	Maximum	Average ± SD	Scott–Knott Groups (5%)
Seed length (mm)	18.9	29.7	24.76 ± 2.16	a–j
Seed width (mm)	15.8	23.6	19.01 ± 1.29	a–g
Seed thickness (mm)	10.1	22.4	13.93 ± 1.51	a–b
Seed weight (g)	2.6	6.8	4.89 ± 0.88	a–i
Water content of seeds (%)	5.7	17.0	11.13 ± 1.88	a–e

SD: standard deviation. Note: Detailed values of the matrices by Scott–Knott group are available in the Appendix A.2 (Table A2).

**Table 5:** Descriptive statistics of physiological quality of seeds and initial development of *Hymenaea martiana* seedlings from 160 mother plants.

Variable	Minimum	Maximum	Average ± SD	Scott–Knott Groups (5%)
First emergence count (%)	0	94	42.00 ± 25.39	a–g
Emergence percentage (%)	8	96	59.00 ± 26.07	a–d
Emergence speed index	0.09	1.24	0.65 ± 0.27	a–h
Mean emergence time (days)	16	30	23.00 ± 2.85	a–j
Length of aerial part (cm)	4.7	27.4	17.73 ± 3.91	a–g
Length of the root system (cm)	8.0	24.7	14.96 ± 3.14	a–e
Dry mass of aerial part (g seedling^−1^)	0.23	16.1	1.01 ± 1.55	a–d
Root dry mass (g seedling^−1^)	0.15	1.32	0.32 ± 0.14	a–e

SD: standard deviation. Note: Detailed values of the matrices by Scott–Knott group are available in the Appendix A.3 (Table A3).

## Data Availability

The original contributions presented in the study are included in the article. The data presented in this study are available on request from the corresponding author.

## Appendix A

Scott-Knott test for the physical characterization of fruits and seeds and the physiological quality of *Hymenaea martiana* seeds.

### Appendix A.1

**Table A1:** Physical characterization of *Hymenaea martiana* fruits from 160 mother plants.

**Group ***	**Averages**	**Mother Plants ****
	**Fruit length (mm)**	
Group 1 (a)	153.27	23
Group 2 (b)	134.52	152
Group 3 (c)	119.55–124.42	76, 9, 59 and 68
Group 4 (d)	115.45–116.32	56, 74, 153 and 8
Group 5 (e)	107.10–112.30	21, 88, 17, 5, 62, 143, 129, 126, 33, 65, 71, 86 and 75
Group 6 (f)	99.20–105.67	131, 101, 100, 107, 134, 43, 69, 142, 39, 123, 29, 66, 85, 48, 125 and 40
Group 7 (g)	92.82–98.42	136, 106, 111, 27, 70, 16, 114, 36, 132, 102, 51, 80, 2, 141, 121, 31, 79, 22, 42, 34, 44, 26, 133, 146, 120, 95, 63, 64, 130, 99, 117 and 67
Group 8 (h)	86.50–92.17	15, 81, 135, 3, 4, 156, 119, 50, 25, 41, 104, 108, 60, 32, 103, 122, 61, 115, 28, 137, 148, 24, 72, 38, 20, 37, 96 and 154
Group 9 (i)	78.80–86.02	7, 124, 97, 11, 151, 160, 105, 157, 116, 149, 147, 84, 10, 55, 6, 46, 35, 57, 118, 30, 19, 49, 18, 77, 113, 12, 47, 87, 144, 128, 90, 109, 158 and 45
Group 10 (j)	71.82–77.45	159, 110, 140, 150, 127, 78, 82, 89, 145, 73, 112, 14, 98 and 1
Group 11 (k)	59.42–70.12	52, 139, 94, 54, 93, 155, 138, 91, 53, 83, 92, 13 and 58
	**Fruit width (mm)**	
Group 1 (a)	58.15–62.50	66, 39, 74, 152 and 151
Group 2 (b)	52.50–57.55	68, 88, 5, 56, 59, 8, 23, 100, 17, 9 and 48
Group 3 (c)	47.72–51.80	117, 49, 143, 16, 65, 113, 125, 35, 76, 36, 137, 102, 71, 142, 153, 126, 31, 21, 86, 85, 77, 44, 101, 75, 129 and 42
Group 4 (d)	43.62–47.17	80, 27, 38, 62, 149, 109, 78, 41, 114, 120, 20, 123, 19, 29, 84, 2, 11, 60, 106, 40, 10, 43, 131, 130, 45, 50, 33, 63, 26, 95, 132, 104, 108, 148, 18, 107, 79, 32 and 67
Group 5 (e)	37.80–43.22	4, 3, 118, 83, 93, 103, 13, 55, 91, 145, 111, 57, 1, 82, 128, 127, 64, 58, 6, 136, 150, 119, 90, 122, 51, 34, 133, 158, 25, 28, 98, 146, 47, 24, 30, 134, 121, 12, 81, 99, 37, 89, 115, 69, 46, 96, 61, 22 and 87
Group 6 (f)	33.27–37.37	70, 138, 154, 54, 52, 147, 73, 156, 140, 116, 97, 112, 7, 124, 92, 144, 157, 105, 14, 53, 110, 141 and 15
Group 7 (g)	29.65–31.92	132, 139, 160 and 72
Group 8 (h)	26.90–28.57	94, 159 and 155
	**Fruit thickness (mm)**	
Group 1 (a)	35.50–39.80	79, 100, 117, 50, 35, 63, 143, 101, 125, 48 and 23
Group 2 (b)	32.00–34.90	137, 1, 19, 135, 121, 115, 18, 98, 90, 112, 130, 74, 60, 111, 87, 17, 114, 129, 42, 47, 59, 44, 109,85, 113, 6 and 43
Group 3 (c)	29.50–31.80	34, 122, 64, 75, 142, 84, 58, 56, 41, 128, 7, 14, 11, 39, 31, 95, 120, 2, 134, 110, 20, 78, 93, 51, 73, 108, 83, 80, 106, 92, 148, 65, 10, 57, 102, 26, 127 and 88
Group 4 (d)	26.90–29.42	53, 68, 15, 140, 30, 152, 16, 86, 37, 151, 28, 67, 55, 139, 9, 138, 52, 8, 76, 107, 149, 69, 77, 96, 24, 131, 36, 62, 158, 22, 126, 27, 21, 124, 123, 136 and 38
Group 5 (e)	22.17–26.77	25, 71, 103, 147, 144, 33, 66, 150, 29, 13, 40, 157, 146, 89, 133, 5, 99, 54, 91, 81, 145, 61, 82, 3, 4, 12 and 104
Group 6 (f)	18.65–21.92	154, 70, 116, 141, 49, 105, 119, 32, 153, 132, 97, 118 and 72
Group 7 (g)	14.07–18.10	159, 160, 155, 46, 94, 156 and 45
	**Fruit weight (g)**	
Group 1 (a)	140.92	23
Group 2 (b)	123.11–128.52	68 and 152
Group 3 (c)	103.85–112.89	48, 74 and 153
Group 4 (d)	93.31–98.28	8, 88, 39, 59, 141 and 9
Group 5 (e)	67.42–83.91	102, 35, 40, 101, 154, 72, 108, 146, 69, 66, 43, 107, 61, 42, 63, 78, 17, 21, 67, 31, 103, 64, 156, 76, 36, 71, 44, 75, 123, 142, 80, 100, 143, 70, 5, 62, 85, 33, 56, 109, 129 and 86
Group 6 (f)	47.28–66.35	160, 81, 149, 99, 157, 25, 18, 11, 96, 147, 84, 15, 119, 28, 3, 4, 37, 128, 133, 24, 111, 34, 132, 113, 134, 144, 16, 120, 97, 90, 10, 145, 49, 77, 38, 135, 151, 117, 6, 27,12, 2, 118, 22, 105, 98, 122, 47, 60, 158, 115, 26, 79, 30, 20, 51, 114, 148, 29, 116, 104, 106, 131, 95, 19, 41, 127, 87, 32, 65, 45, 50, 130, 126, 137 and 121
Group 7 (g)	37.74–45.46	112, 92, 73, 89, 93, 1, 46, 82, 13, 124, 150, 57, 7, 136, 155, 14, 55 and 159
Group 8 (h)	17.94–35.87	139, 54, 52, 53, 138, 58, 91, 94, 83, 140 and 110
	**Number of seeds per fruit**	
Group 1 (a)	9.50	23
Group 2 (b)	5.75–6.50	76, 71, 103, 86, 59 and 154
Group 3 (c)	4.75–5.25	40, 72, 36, 141, 125, 62, 146, 115, 114 and 31
Group 4 (d)	3.75–4.50	116, 42, 81, 60, 6, 131, 35, 144, 33, 128, 121, 68, 69, 75, 61, 157, 156, 153, 95, 105, 143, 88, 160, 158, 85, 5, 8, 9, 70, 152, 90, 109, 51, 74, 107, 110, 20, 48, 80 and 123
Group 5 (e)	2.75–3.50	124, 151, 26, 29, 126, 1 9, 91, 11, 140, 12, 77, 148, 14, 37, 56, 50, 94, 67, 78, 21, 120, 43, 119, 113, 106, 30, 136, 10, 101, 46, 15, 84, 117, 111, 41, 145, 39, 118, 98, 134, 132, 22, 66, 34, 17, 28, 142, 18, 27, 135, 133, 63, 64, 102, 92, 159, 47, 147, 97 and 96
Group 6 (f)	1.50–2.50	89, 93, 53, 73, 127, 58, 65, 82, 138, 79, 16, 139, 150, 149, 112, 137, 99, 52, 155, 130, 129, 38, 49, 54, 25, 108, 7, 104, 1, 3, 4, 87, 83, 122, 13, 45, 44, 100, 57, 55, 32, 24 and 2

* The groups for each variable were classified according to the Scott–Knott test at 5% probability, with higher values found in the groups closest to the letter “a”. ** *H. martiana* trees from Areia—PB (1–136), Bananeiras—PB (137–140); Cuité—PB (141); Marcação—PB (142–143); Nova Floresta—PB (144–156); Jaçanã—RN (157–160).

### Appendix A.2

**Table A2:** Physical characterization of *Hymenaea martiana* seeds from 160 mother plants.

**Group ***	**Averages**	**Mother Plants ****
	**Seed length (mm)**	
Group 1 (a)	29.22–29.67	35, 5 and 21
Group 2 (b)	28.05–28.75	67, 2, 39, 36, 48 and 68
Group 3 (c)	26.70–27.82	33, 133, 152, 20, 69, 76, 60, 56, 77, 102, 104, 106, 59, 29, 70, 86, 84, 66, 153, 75, 137, 74 and 65
Group 4 (d)	25.50–26.42	72, 109, 22, 32, 10, 42, 19, 88, 11, 143, 142, 71, 16, 100, 41, 116, 17, 156, 31, 4, 3, 78, 119, 12, 125, 23, 63, 118, 37, 146, 62, 141 and 61
Group 5 (e)	24.60–25.42	40, 27, 28, 151, 101, 117, 114, 107, 120, 24, 155, 58, 158, 113, 159, 134, 79, 135, 38, 9, 26, 44, 126 and 49
Group 6 (f)	23.80–24.52	147, 45, 121, 111, 148, 8, 64, 81, 47, 13, 94, 103, 30, 25, 154, 122, 131, 115, 18, 50 and 127
Group 7 (g)	22.95–23.70	95, 150, 15, 90, 132, 85, 7, 96, 149, 34, 98, 136, 52, 99 and 123
Group 8 (h)	21.45–22.75	6, 93, 57, 73, 140, 97, 82, 55, 112, 138, 87, 80, 53, 14, 46, 110, 124, 105, 108, 128, 139, 145, 91 e 160
Group 9 (i)	20.30–21.35	43, 144, 130, 92, 89, 54, 129, 83 and 157
Group 10 (j)	18.90–19.75	1 and 51
	**Seed width (mm)**	
Group 1 (a)	23.60	125
Group 2 (b)	22.37	35
Group 3 (c)	20.65–21.78	158, 36, 106, 109, 11, 58, 10, 74, 2, 19, 48 and 12
Group 4 (d)	19.72–20.55	3, 4, 134, 137, 154, 111, 21, 49, 47, 7, 115, 39, 84, 156, 143, 44, 153, 142, 135, 126, 114, 23, 65, 63, 60, 56, 94, 68, 118, 141, 103, 121 and 13
Group 5 (e)	19.00–19.67	151, 5, 22, 57, 76, 81, 69, 123, 83, 73, 53, 80, 37, 112, 95, 67, 122, 138, 77, 127, 110, 104, 75, 107, 15, 88, 133, 59, 101, 62, 42, 146, 155, 117, 50 and 116
Group 6 (f)	17.82–18.95	86, 97, 132, 91, 6, 150, 128, 9, 92, 33, 25, 139, 124, 66, 40, 72, 31, 30, 105, 45, 136, 52, 149, 147, 98, 102, 78, 24, 38, 131, 140, 64, 16, 113, 18, 108, 17, 152, 90, 34, 28, 148, 70, 41, 120, 61, 85, 26 and 29
Group 7 (g)	15.77–17.75	89, 93, 130, 82, 157, 160, 46, 99, 32, 96, 159, 20, 71, 55, 8, 14, 129, 51, 79, 145, 54, 144, 100, 119, 1, 43, 87 and 27
	**Seed thickness (mm)**	
Group 1 (a)	13.92–22.37	138, 59, 79, 55, 140, 132, 124, 14, 74, 17, 41, 95, 1, 28, 131, 117, 66, 65, 104, 45, 139, 78, 136, 39, 7, 150, 5, 34, 118, 35, 13, 144, 63, 127, 155, 113, 33, 10, 15, 29, 137, 135, 88, 120, 101, 112, 111, 37, 133, 57, 107, 77, 108, 44, 151, 156, 67, 21, 16, 126, 3, 4, 141, 48, 73, 148, 50, 54, 134, 53, 56, 49, 2, 19, 68, 12, 122, 11, 106, 94, 149, 58 and 36
Group 2 (b)	10.15–13.85	89, 23, 103, 92, 110, 115, 154, 93, 42, 90, 128, 87, 114, 71, 43, 157, 158, 31, 160, 60, 27, 86, 159, 51, 47, 109, 62, 32, 97, 72, 121, 105, 81, 82, 102, 98, 40, 70, 30, 8, 6, 9, 22, 83, 24, 61, 69, 20, 76, 25, 147, 116, 119, 26, 91, 80, 129, 123, 64, 75, 46, 125, 38, 130, 145, 85, 146, 18, 96, 100, 52, 99, 152, 153 and 84
	**Seed weight (g)**	
Group 1 (a)	6.36–6.85	138, 58, 19, 125, 21, 106, 12, 11, 68, 2 and 67
Group 2 (b)	5.85–6.29	65, 156, 94, 29, 153, 35, 5, 118, 39, 3, 4, 56, 74, 141, 10 e 48
Group 3 (c)	5.47–5.73	17, 75, 88, 134, 44, 122, 61, 133, 63, 77 and 126
Group 4 (d)	5.03–5.42	38, 116, 101, 22, 151, 66, 155, 120, 107, 111, 41, 123, 36, 142, 104, 70, 16, 100, 146, 84, 59, 37, 127, 76, 33, 13, 78, 152 and 50
Group 5 (e)	4.64–4.98	149, 34, 42, 147, 45, 7, 60, 24, 27, 25, 64, 85, 131, 53, 62, 86, 117, 28, 95, 31, 26, 143, 15, 148, 69, 113, 72, 109 and 43
Group 6 (f)	4.17–4.61	32, 83, 98, 139, 124, 119, 114, 140, 80, 18, 135, 49, 136, 71, 121, 150, 108, 52, 144, 40, 47, 20, 81, 79, 102, 30, 9, 112, 73 and 57
Group 7 (g)	3.66–4.15	54, 46, 128, 129, 157, 55, 90, 97, 159, 99, 14, 115, 103, 6, 96, 137, 8, 105, 154, 145, 132 and 23
Group 8 (h)	3.22–3.58	92, 160, 93, 158, 130, 87, 1, 82 and 110
Group 9 (i)	2.62–3.07	89, 51 and 91
	**Water content of seeds (%)**	
Group 1 (a)	14.70–16.96	73, 98, 118, 119 and 41
Group 2 (b)	12.59–14.11	91, 138, 75, 48, 111, 106, 25, 9, 134, 74, 69, 7, 110, 114, 137, 68, 77, 51, 19, 125, 115, 136, 58, 76, 63, 78, 131, 2, 105, 86, 87, 15, 133, 18, 64, 21, 16 and 132
Group 3 (c)	10.85–12.48	79, 53, 54, 121, 82, 33, 12, 103, 70, 104, 61, 81, 31,109, 42,40, 101, 30, 117, 116, 27, 11, 36, 6, 38, 20, 14, 141, 102, 100, 8, 60, 57, 67, 26, 112, 3, 4, 13,52, 5, 80. 23, 92, 113, 130, 66, 65, 37, 135, 90, 62, 59 and 56
Group 4 (d)	9.09–10.77	140, 127, 129, 46, 126, 157, 45, 55, 139, 160, 108, 95, 89, 94, 123, 156, 29, 1, 88, 50, 84, 72, 159, 158, 154, 47, 28, 145, 24, 10, 39, 71, 17, 32 and 22
Group 5 (e)	5.73–8.93	153, 148, 43, 128, 96, 147, 120, 44, 35, 155, 149, 83 e 107, 150, 144, 49, 34, 146, 85, 122, 99, 124, 151, 97, 93 and 152

* The groups for each variable were classified according to the Scott–Knott test at 5% probability, with higher values found in the groups closest to the letter “a”. ** *H. martiana* trees from Areia—PB (1–136), Bananeiras—PB (137–140); Cuité—PB (141); Marcação—PB (142–143); Nova Floresta—PB (144–156); Jaçanã—RN (157–160).

### Appendix A.3

**Table A3:** Physiological quality of seeds and initial development of *Hymenaea martiana* seedlings from 160 mother plants.

**Group ***	**Averages**	**Mother Plants ****
	**First emergence count (%)**	
Group 1 (a)	77.0–94.0	124, 111, 150, 40, 157, 86, 158, 159, 138, 152, 106, 97, 120, 141, 96, 103 and 160
Group 2 (b)	64.0–75.0	122, 72, 105, 32, 119, 19, 144, 151, 74, 54, 56, 57, 55, 156, 136, 128,110, 1, 42, 114, 78, 148 and 81
Group 3 (c)	51.0–61.0	60, 35, 155, 108, 82, 58, 116, 113, 135, 133, 59, 125, 52, 146, 93, 121, 112, 2, 48, 23, 73, 94, 104, 99 and 41
Group 4 (d)	40.0–50.0	123, 145, 118, 131, 77, 153, 126, 101, 91, 49, 154, 149, 134, 140, 53, 79, 107, 26, 44, 84, 80, 83, 34, 85 and 147
Group 5 (e)	26.0–38.0	29, 98, 20, 95, 39, 43, 63, 100, 132, 33 and 47
Group 6 (f)	15.0–25.0	66, 13, 18, 127, 4, 36, 27, 22, 16, 71, 25, 115, 51, 17, 8, 90, 117, 92, 3, 69, 15, 14, 64, 129, 102, 38, 46, 9, 45, 76, 31, 37 and 75
Group 7 (g)	0.0–14.0	11, 12, 7, 88, 137, 24, 10, 28, 67, 89, 68, 65, 62, 130, 50, 143, 87, 21, 142, 5, 30, 139, 70, 61 and 8
	**Emergence percentage (%)**	
Group 1 (a)	74.0–96.0	99, 1, 54, 6, 125, 19, 148, 132, 140, 91, 93, 134, 40, 86, 150, 157, 123, 151, 159, 135, 105, 78, 97, 104, 107, 73, 108, 138, 80, 111, 120, 124, 81, 158, 106, 152, 96, 103, 160 and 141
Group 2 (b)	83.0–73.0	83, 147, 3, 58, 82, 16, 43, 149, 77, 26, 84, 13, 116, 155, 4, 59, 28, 146, 44, 121, 113, 60, 52, 35, 49, 61, 85, 17, 2, 45, 27, 41, 129, 48, 75, 131, 23, 34, 130, 119, 118, 112, 122, 74, 5, 127, 136, 126, 70, 57, 144, 94, 100, 22, 32, 156, 72, 71, 38, 42, 56, 110, 109, 114, 128, 55 and 133
Group 3 (c)	31.0–50.0	88, 95, 9, 20, 37, 65, 63, 30, 33, 25, 47, 64, 24, 29, 98, 46, 18, 137, 153, 115, 50, 154, 14, 90, 69, 39, 66, 53, 145, 79, 101, 15, 68 and 139
Group 4 (d)	8.0–30.0	87, 21, 7, 10, 11, 89, 62, 36, 8, 51, 102, 92, 67, 76, 31, 12, 117, 142 and 143
	**Emergence speed index**	
Group 1 (a)	1.23–1.24	103, 160 and 120
Group 2 (b)	1.04–1.14	152, 96, 97, 157, 86, 158, 106, 74, 159 and 141
Group 3 (c)	0.89–1.02	6, 104, 57, 73, 1, 132, 134, 133, 150, 151, 42, 144, 78, 40, 124, 136, 111, 148, 81, 105 and 138
Group 4 (d)	0.65–0.88	82, 35, 71, 85, 113, 38, 34, 127, 155, 58, 100, 126, 122, 118, 52, 112, 75, 91, 60, 140, 94, 121, 146, 59, 99, 131, 2, 93, 32, 41, 48, 128, 125, 72, 123, 23, 56, 109, 80, 114, 119, 107, 19, 135, 110, 156, 108, 54 and 55
Group 5 (e)	0.52–0.64	26, 16, 28, 3, 4, 13, 43, 154, 53, 77, 61, 79, 259, 83, 27, 17, 5, 45, 130,84, 129, 49, 116, 70, 44, 147 and 22
Group 6 (f)	0.39–0.51	95, 46, 18, 29, 50, 115, 14, 63, 90, 66, 68, 33, 139, 39, 153, 101, 15, 47, 98, 145 and 69
Group 7 (g)	0.26–0.38	76, 143, 88, 142, 117, 31, 30, 9, 37, 24, 65, 137, 20, 25 and 64
Group 8 (h)	0.09–0.25	87, 7, 21, 11, 10, 89, 51, 67, 62, 36, 8, 12, 102 and 92
	**Mean emergence time (days)**	
Group 1 (a)	28.3–29.7	28, 24, 5, 12, 11 and 137
Group 2 (b)	26.2–27.9	14, 142, 66, 50, 89, 129, 17, 16, 13, 18, 45, 143, 38, 10, 7, 30, 26, 4, 27, 88, 71, 61, 139, 70, 68, 130, 22 and 67
Group 3 (c)	24.8–26.0	108, 37, 15, 107, 126, 43, 25, 77, 39, 123, 140, 3, 91, 64, 127, 115, 46, 80, 90 and 100
Group 4 (d)	23.5–24.6	35, 94, 149, 51, 104, 118, 85, 53, 73, 99, 65, 44, 117, 122, 29, 9, 93, 135, 101, 34 and 49
Group 5 (e)	22.4–23.3	6, 78, 20, 96, 151, 128, 112, 116, 36, 113, 83, 111, 81, 76, 92, 145, 125, 124 and 84
Group 6 (f)	21.3–22.3	155, 141, 131, 134, 138, 31, 87, 69, 21, 105, 114, 56, 98, 102, 62, 75, 153, 19, 150, 72, 32, 152 and 109
Group 7 (g)	19.6–21.0	154, 119, 42, 146, 121, 148, 57, 95, 157, 58, 63, 41, 133, 97, 40, 48, 23, 2, 156, 1, 33, 106, 54, 147, 8, 60, 79, 55, 82, 110, 158, 132 and 52
Group 8 (h)	18.5–19.3	136, 86, 160, 159, 144, 103, 47 and 59
Group 9 (i)	17.1	120
Group 10 (j)	15.5	74
	**Length of aerial part (cm)**	
Group 1 (a)	23.40–27.42	36, 156, 96, 54, 55, 104, 77, 133, 56, 52 and 74
Group 2 (b)	20.75–22.95	101, 33, 31, 119, 141, 86, 128, 122, 134, 97, 144, 73, 32, 102, 2, 121, 42, 53, 29, 59, 120, 58, 106, 99, 40, 131, 158 and 103
Group 3 (c)	18.75–20.50	160, 154, 157, 148, 123, 81, 82, 41, 1, 94, 107, 152, 91, 47, 75, 63, 17, 108, 159, 153 and 19
Group 4 (d)	14.75–18.47	100, 145, 68, 7, 98, 85, 105, 113, 46, 14, 45, 37, 80, 25, 8, 139, 49, 83, 48, 22, 79, 110, 69, 51, 142, 92, 124, 151, 95, 27, 43, 140, 87, 84, 93, 9, 44, 132, 109, 114, 35, 78, 150, 38, 57, 125, 149, 21, 111, 88, 62, 60, 155, 138, 146, 20, 72, 34, 136, 76, 127, 116, 147 and 65
Group 5 (e)	12.17–14.55	115, 30, 90, 71, 129, 64, 4, 15, 16, 130, 18, 135, 28, 70, 70, 118, 50, 26, 11, 61, 3, 6, 5, 143 and 66
Group 6 (f)	10.02–11.92	10, 117, 67, 137, 39, 13, 12, 126, 24, 89 and 112
Group 7 (g)	4.72	23
	**Length of the root system (cm)**	
Group 1 (a)	23.77–24.72	19, 103, 119 and 1
Group 2 (b)	20.42–22.30	131, 102, 33, 121, 160, 125, 53, 133 and 23
Group 3 (c)	19.97–16.75	43, 83, 97, 84, 4, 129, 80, 122, 57, 17, 58, 94, 127, 147, 132, 140, 128, 51, 120, 81, 90 and 2
Group 4 (d)	13.45–16.50	18, 54, 155, 105, 139, 150, 107, 5, 55, 126, 146, 159, 118, 74, 85, 156, 72, 13, 112, 34, 108, 7, 50, 148, 49, 79, 71, 26, 136, 135, 70, 100, 154, 44, 37, 25, 114, 96, 113, 64, 35, 93, 89, 9, 142, 109, 92, 27, 14, 24, 124, 6, 77, 111, 3, 78, 145, 45, 144, 20, 86, 115, 141, 101, 8, 41, 42, 32, 40, 106, 134, 66, 82, 38 and 110
Group 5 (e)	8.00–13.22	10, 21, 104, 123, 30, 95, 88, 117, 39, 62, 29, 87, 65, 63, 60, 48, 130, 31, 11, 68, 36, 91, 52, 16, 56, 138, 99, 157, 67, 69, 75, 12, 152, 47, 149, 153, 158, 116, 143, 59, 76, 15, 28, 61, 46, 73, 137, 98, 151 and 22
	**Dry mass of aerial part (g seedling^−1^)**	
Group 1 (a)	16.12	34
Group 2 (b)	12.77	23
Group 3 (c)	0.85–1.67	20, 149, 29, 152, 62, 44, 159, 61, 99, 95, 31, 65, 21, 69, 97, 111, 108, 114, 71, 53, 36, 85, 70, 146, 127, 145, 148, 128, 96, 144, 72, 116, 60, 136, 47, 113, 77, 52, 101, 122, 41, 84, 76, 104, 32, 35, 42, 94, 48, 57, 119, 125, 131, 40, 158, 134, 54, 153, 73, 156, 56, 56, 58, 59, 86, 63, 103, 2, 19, 75, 121, 33, 106, 79, 141,133, 120 and 74
Group 4 (d)	0.23–0.84	24, 13, 89, 67, 137, 7, 30, 98, 66, 105, 26, 117, 90, 28, 16, 130, 3, 110, 139, 6, 5, 11, 12, 100, 50, 126, 143, 115, 27, 140, 22, 135, 88, 49, 46, 9, 14, 118, 93, 82, 129, 45, 112, 102, 18, 93, 82, 129, 45, 112, 102, 18, 15, 38, 25, 51, 80, 43, 87, 4, 39, 151, 107, 8, 91, 160, 157, 17, 68, 92, 37, 124, 138, 123, 78, 109, 1, 147, 10, 83, 154, 55, 132, 150, 81, 64, 155 and 142
	**Root dry mass (g seedling^−1^)**	
Group 1 (a)	1.31	23
Group 2 (b)	0.63–0.80	2, 87, 119, 19, 34 and 102
Group 3 (c)	0.41–0.56	94, 113, 103, 73, 114, 59, 132, 68, 116, 40, 14, 72, 48, 147, 36, 44, 86, 120, 33, 42, 41, 121, 141, 106 and 58
Group 4 (d)	0.28–0.40	122, 81, 20, 61,75, 37, 109, 8, 54, 92, 112, 130, 71, 51, 39, 142, 60, 85, 143, 25, 83, 118, 88, 35, 91, 156, 9, 77, 101, 4, 84, 70, 127, 145, 104, 47, 80, 125, 56, 74, 96, 66, 136, 43, 134, 160, 155, 131, 154, 57, 144, 153, 133, 148 and 63
Group 5 (e)	0.15–0.27	7, 89, 139, 82, 124, 49, 46, 98, 55, 22, 90, 117, 45, 110, 30, 93, 28, 126, 18, 100, 69, 26, 95, 29, 79, 12, 52, 32, 13, 78, 99, 11, 137, 123, 50, 27, 157, 159, 128, 151, 53, 149, 67, 76, 115, 6, 111, 16, 62, 3, 107, 150, 129, 65, 38, 146, 64, 24, 31, 1, 21, 15, 5, 105, 138, 158, 152, 97, 140, 10, 108, 17 and 135

* The groups of each variable were classified according to the Scott–Knott test at 5% probability, in which higher values were found in the groups closest to the letter “a”. ** *H. martiana* trees from Areia—PB (1–136), Bananeiras—PB (137–140); Cuité—PB (141); Marcação—PB (142–143); Nova Floresta—PB (144–156); Jaçanã—RN (157–160).
